# Predicting the unexpected in stomatal gas exchange: not just an open-and-shut case

**DOI:** 10.1042/BST20190632

**Published:** 2020-05-26

**Authors:** Martina Klejchová, Adrian Hills, Michael R. Blatt

**Affiliations:** Laboratory of Plant Physiology and Biophysics, University of Glasgow, Bower Building, Glasgow G12 8QQ, U.K.

**Keywords:** carbon fixation, guard cell, mathematical modelling, stomata, water use efficiency

## Abstract

Plant membrane transport, like transport across all eukaryotic membranes, is highly non-linear and leads to interactions with characteristics so complex that they defy intuitive understanding. The physiological behaviour of stomatal guard cells is a case in point in which, for example, mutations expected to influence stomatal closing have profound effects on stomatal opening and manipulating transport across the vacuolar membrane affects the plasma membrane. Quantitative mathematical modelling is an essential tool in these circumstances, both to integrate the knowledge of each transport process and to understand the consequences of their manipulation *in vivo*. Here, we outline the OnGuard modelling environment and its use as a guide to predicting the emergent properties arising from the interactions between non-linear transport processes. We summarise some of the recent insights arising from OnGuard, demonstrate its utility in interpreting stomatal behaviour, and suggest ways in which the OnGuard environment may facilitate ‘reverse-engineering’ of stomata to improve water use efficiency and carbon assimilation.

## Introduction

Understanding and predicting stomatal behaviour is vital to inform agricultural practices and potentially anticipate plant responses to climatic changes [1]. Stomata are pores found on the leaf epidermis that form between pairs of specialised cells, the guard cells. Stomata allow the uptake of CO_2_, required for photosynthesis at the expense of water loss via transpiration from the tissues within the leaf. Gas exchange is regulated by exogenous signals, such as CO_2_, water availability, and light [2–12], each of which affects stomatal pore size. Changes in the size and shape of stomatal pores arise from water fluxes that are commonly driven by the accumulation and depletion of osmotically active ions in the guard cells [7]. Therefore, guard cell solute transport and metabolism have a substantial impact on plant fitness and feed directly into the photosynthetic yield and water status of the plant. To better predict and engineer plant physiology, one has to consider the changes occurring at the guard cell level and how these relate to the tissue-wide responses of the leaf. In other words, there is a need to bridge the gap from the microscopic events of cellular ion transport and metabolism to the macroscopic properties of gas exchange and its regulation in the leaf and whole-plant.

Though it often goes unacknowledged, the network of interactions between metabolism, ion transport, and solute partitioning among cellular compartments poses a major barrier to a quantitative understanding of cellular physiology. Nowhere is the challenge of this barrier more evident than in understanding the stomatal regulation of gas exchange in the leaf. The physiology of the stomatal guard cells is dictated by the characteristics intrinsic to each process, whether of ion transport, organic solute synthesis, or its catabolism. Each of these processes incorporates kinetics that are highly non-linear, often with respect to multiple parameter inputs, including substrate concentrations and membrane voltage, as well as regulatory inputs that engage ligand and other post-translational controls. Such non-linearities underpin much of physiology that is seemingly counterintuitive, what is often referred to as the ‘emergent’ behaviours of the guard cells. Thus, even without considering transcriptional and translational regulation, addressing how guard cells respond to environmental inputs, and their coupling to the macroscopic properties of the whole leaf, demands a full and quantitative accounting of the characteristics for each transport, metabolic, and buffering reaction.

## Modelling stomata

A wealth of information exists to address the mechanism of guard cell physiology and stomatal function, both in relation to solute transport [7] and metabolism [13]. The majority of the molecular effectors in transport and metabolism are known, their kinetic properties detailed and, in many cases, their cellular interactions identified. There is also a very large body of information relating stomatal behaviour, measured as foliar gas exchange, with environmental inputs of temperature, relative atmospheric humidity, light, and its consequences for photosynthetic carbon assimilation [9,14]. Thus, expanding our knowledge to uncover emergent behaviours is possible through mathematical approaches to simulate stomatal behaviour and bridge the gap to foliar gas exchange, validating through experiment both at the whole-plant and at the guard cell levels.

In general two different mathematical approaches to stomata have been pursued that divide across scales. The first approach has been to treat stomata as discrete, phenomenological components on the scale of the whole leaf and plant, each stoma serving as a pathway for transpirational water loss and CO_2_ uptake [15–21]. These macroscopic approaches consider stomata as conductance units for gas exchange that vary in quasi-linear or monotonic (hyperbolic) fashion to environmental inputs of light, water availability, atmospheric water vapour, and CO_2_, their sum yielding the stomatal conductance (*g*_s_) of the whole-plant. Indeed, robust models of gas exchange have proven highly successful in describing stomatal transpiration and CO_2_ exchange from leaf to canopy [21–25]. However, they do not capture the cellular components needed to translate *g*_s_ to the molecular mechanics of the guard cell; as a consequence, these approaches lack the links essential for ‘reverse-engineering’ from the whole-plant to the guard cell.

The second approach has focused on modelling the subcellular components of stomata, notably the transporters and their associated signal cascades in the guard cells, that determine the ion, solute and water fluxes for stomatal opening and closing [7,9,26]. Here, one approach, borrowed from the methods of logic circuit design, describes the guard cell in terms of Boolean nodes and links that connect these nodes. The power of Boolean models lies in its use as a tool to analyse networks for which there are a large number of components and possible connections between them but little quantitative information [27–29]. Its most common application is to identify and rank these connections, effectively mapping plausible causalities within a network. Boolean models are defined through logic gates that can only be ‘on’ or ‘off’, which simplifies analysis but omits the kinetics relationships that are essential to understanding dynamic interactions, their temporal associations and emergent behaviours. Sun et al. [30] have applied Boolean network analysis to guard cell signalling, but these outputs are disconnected from any meaningful physiological mechanisms or their temporal kinetics.

Mechanistic models offer the greatest potential for true physiological insights where these models incorporate the kinetic properties of the individual components, encoding these with parameters to define their operation [31]. The hydromechanical model of Buckley et al. [16] represented a step in this direction, proposing a simple hyperbolic relation between the ATP concentration of the guard cells and their osmotic content. Similar approaches have sought to relate stomatal function to hormonal regulation, notably by ABA [21]. These models lack explicit detail of the underlying non-linearities for the individual transport processes, however, and therefore the structure needed to define stomatal function from the essential molecular mechanics [7]. It is important to note, too, that many of the efforts towards modelling have sought analytic solutions for endpoint or stationary states only. As such, these efforts fail to address the wealth of information available relating to the temporal kinetics for stomatal movements and transpiration.

To date, only the OnGuard platform [32–34] has encapsulated the mechanistic components, their parameters, and the intrinsic non-linearities of solute transport sufficient to accurately simulate guard cell physiology and the stomatal movements that it engenders. The latest revision of the OnGuard platform, OnGuard2, also bridges the gap between the macroscopic characteristics of transpirational water relations in the whole-plant and the microscopic behaviour of guard cells in solute and water transport that drives stomatal movements. Most important, much as *in vivo*, the OnGuard platform connects transport at the two key membranes, the tonoplast and plasma membrane, through the core kinetic variables of membrane voltage as well as substrate and regulatory ligand concentrations. The importance of membrane voltage, especially, lies in its role in feedback between transporters and even with respect to a single transporter.

Consider K^+^ transport out of the guard cell. The guard cell outward-rectifying K^+^ channel — in *Arabidopsis*, the GORK channel — is strongly voltage-dependent, activating only at voltages positive of the prevailing K^+^ equilibrium voltage, *E*_K_ [7,35,36]. Depolarising the membrane activates the channel for K^+^ efflux from the cell, but this activity is countered by the K^+^ flux itself. As K^+^ passes outward through the channel, across the plasma membrane, it carries charge to repolarize the membrane. In other words, even without any effects from other regulatory processes, the activity of the channel counteracts its own flux as determined by intrinsic kinetic relations of the channel gate. Of course, the membrane voltage will be affected by every other transporter that moves a net charge across the membrane. The consequence is that, in the steady-state, ion transport is a highly non-linear process that is not solely determined by the gating characteristics of GORK *per se*, but by the balance of ion and charge transport across the membrane as a whole.

## The elements of the OnGuard platform

Stomatal movements are ideally suited to a ‘bottom-up’ approach in mathematical modelling. They are governed by quantitative relations that describe mass and charge conservation, the ion gradients and permeabilities across each membrane, and the drivers of ion and water flux [7,37,38]. These physico-chemical relations are easy to incorporate mathematically and they constrain all homeostatic interactions within any model. For plant cells, and especially for the guard cell, the important output variables are the cell volume and osmolality, water potential and turgor, the voltages across each membrane, the various ion concentrations, and the corresponding ion fluxes through each transporter.

The biophysical relations of membrane transport are well-defined and, for guard cells, have been detailed to an extent sufficient for quantitative description. For example, H^+^ transport via ATP-driven pumps and coupled transporters as well as the transport of other ions via channels at the plasma membrane have been characterised, with detailed information on stoichiometry and mechanism [7]. Thus, their operation can be described quantitatively within sets of kinetic equations fully constrained by experimental results. Even if our knowledge of individual transporters at the tonoplast is less well-developed, there is ample data from experiments to define the vacuolar ion contents and fluxes [7,39–43], thereby constraining parameter values needed to comply with experimental results. Essential kinetic data are available also for transport regulation, notably its control by cytosolic-free [Ca^2+^] ([Ca^2+^]_i_) and pH, and in many cases by reactive oxygen species (ROS), and protein phosphorylation [7,44–46].

Of course, there are gaps in our knowledge of many of these transporters, at least their molecular identities and some details of their regulation. However, it is sufficient to know the kinetic relationships that describe a process, even if the genes are not known or the detailed regulatory mechanics have not been resolved. For example, we do not know the proportions of inward-rectifying K^+^ channels that are composed of KAT1 and of KAT2 subunits in *Arabidopsis*, nor under what circumstances the KC1 subunit might also assemble together with KAT1 and KAT2 to form the functional channels [47,48]. Nevertheless, we know how the ensemble K^+^ current is gated by voltage and that its amplitude depends on extracellular [K^+^]; furthermore, we can describe these dependencies in quantitative terms [49–52]. This knowledge is sufficient to describe both dependencies with sufficient detail to model the behaviour of the current *in vivo*. Indeed, knowledge of the different subunits alone is not useful in this context. Similarly, we know that [Ca^2+^]_i_ inactivates the inward-rectifying K^+^ channels of the guard cell [53,54], and surmise that this may occur via phosphorylation by one or more Ca^2+^-dependent protein kinases [55–58]. Quantitative kinetic information is still lacking to model the steps between Ca^2+^ binding, the kinase cascades, and their ultimate phosphorylation of the K^+^ channels. Nevertheless, we know the relationship between [Ca^2+^]_i_ and K^+^ channel activity, and we can safely place the mechanistic details in a mathematical description that subsumes the intermediate kinetics. In effect, such a phenomenological approach introduces modules with adjustable levels of resolution that may be expanded if, and when, studies come to focus on a specific module [31].

## Expanding the OnGuard platform to define and model plant water relations

To bridge scales from the guard cell to whole-plant water relations, OnGuard2 integrates three additional sets of variables and parameters associated with the water relations of the leaf. These additions are sufficient to connect guard cell solute transport and metabolism with water feed from the xylem and transpiration from the leaf to the atmosphere [8]. Water vapour in the substomatal cavity must equilibrate with water in the guard cell wall. Water in the wall, in turn, affects the osmotic potential and ionic activities—that is, their effective concentrations—in the apoplast, and thus impacts on ion and water flux across the guard cell plasma membrane. OnGuard2 determines the partial pressure of water vapour in the substomatal cavity from the gradient in its partial pressures between the sites of evaporation in the leaf and the atmosphere outside. To accommodate water delivery to the leaf, OnGuard2 defines water flux through the xylem as it affects the evaporative surface area within the leaf in relation to the area of the stomatal pore. Finally, OnGuard2 introduces a finite hydraulic permeability to the guard cell plasma membrane in order to place water flux under control of relevant cellular signal cascades, notably [Ca^2+^]_i_ and cytosolic pH [59–62]. These descriptors are sufficient to simulate the behaviour of stomata with changes in atmospheric relative humidity (%RH) and temperature, thereby connecting aperture, and hence stomatal conductance, to the rate of transpiration from the leaf. They give the user direct control over water availability to the leaf, allowing simulations for example of drought conditions as well as the effects of changing atmospheric water vapour pressure. The resulting models accurately predict the effects of leaf and whole-plant transpiration on the molecular processes of guard cell membrane transport and, conversely, they have correctly predicted how manipulating membrane transport affects stomatal conductances of the leaf and whole plant [8].

While iterative computational modelling, such as used by the OnGuard platform, does not pre-define a final endpoint, it does require a starting point — a reference state — from which time increments may be calculated and the dynamics of the model can evolve based on the constraining physical laws, the equations and their parameters that define the components of the model and, most importantly, the interactions that arise from their functioning over time. To aid in resolving such a starting (or reference) state, the OnGuard platform incorporates a Reference State Wizard. The Wizard allows the user to specify the underlying biophysical status of the system and then query the model for solute and metabolic fluxes in total and through each of the model processes in order to establish a starting or Reference State. Obviously establishing a Reference State implies prior knowledge of the probable unit densities, or at least the typical amplitudes of each current, and the characteristic parameters defining the kinetics for each transporter. The biological validity of a model is first judged by this knowledge, which is critical if a model is to avoid indetermination. There is no absolute rule here, but experience sets some basic guidelines, including the need for at least two flux pathways for each solute species if flux balance is to be achieved across a membrane [33]. If these conditions are met, and kinetic detail is available to define quantitatively at least 80–85% of the total flux of all species between compartments, then parameters for the remaining fluxes generally will be constrained sufficiently to render a model with true predictive power. Models based on the Reference States and diurnal Reference Cycles for guard cells of *Vicia* [33,34] and *Arabidopsis* [8,32,63–65] are available for download with the OnGuard platform (www.psrg.org.uk) and a full set of descriptions for the transporters will be found in these publications and in the extended set of tables in the review by Jezek and Blatt [7].

## Interrogating OnGuard platform outputs

Interpreting the output of an OnGuard2 simulation requires the user to interrogate the model variables. Changes in each of these variables — for example, solute concentration, the associated rates of ion and solute flux through each of the transporters, the membrane voltages, [Ca^2+^]_i_ and pH — depend on interactions between the transporters, cytosolic and vacuolar buffering for pH and [Ca^2+^]_I_, and metabolism, just as they do *in vivo*. So, in general, the task of interpretation reduces to one of tracing the sequence of events following a trigger, or change in a specific model parameter, through the network of interrelated homeostatic processes. Here, the output variables, their kinetics, flux, and metabolic origins are most useful and will help in identifying emergent behaviours of the system that are often the most informative aspects of any modelling effort.

Of course, no model is useful unless it yields predictions that are experimentally testable. Simply reproducing known behaviours is generally less informative and cannot validate the modelling effort. There are many predictions that can be drawn from OnGuard2 simulations, a number of which have been validated experimentally. Of these, OnGuard2 models of *Arabidopsis* have accurately predicted the transient stimulation of inward-rectifying K^+^ channels on recovery from steps in ambient %RH, an acceleration in the recovery of stomatal conductance with %RH [8], and co-ordinated alterations in osmotic solute transport across the tonoplast and its retention in the *slac1* mutant [8,66]. Similarly, the OnGuard platform predicted, as a target for enhancing stomatal gas exchange and water use efficiency (defined as the amount of carbon fixed in photosynthesis divided by the amount of water lost via stomatal transpiration), the importance of introducing new channel gating behaviours over simple increases in the populations of channels and pumps at the guard cell plasma membrane [67]. This prediction proved correct in relation to increases in both the number of guard cell K^+^ channels and H^+^-ATPases [68]. It has since been borne out, too, in the first application of optogenetics in plants, demonstrating the efficacy of introducing a synthetic, light-activated K^+^ channel to enhance water use efficiency and carbon assimilation [69]. There are many other predictions that still remain to be tested experimentally, not least among these the effects of changes in %RH on K^+^, anion, Ca^2+^, and other fluxes at the tonoplast.

**Figure 1.**
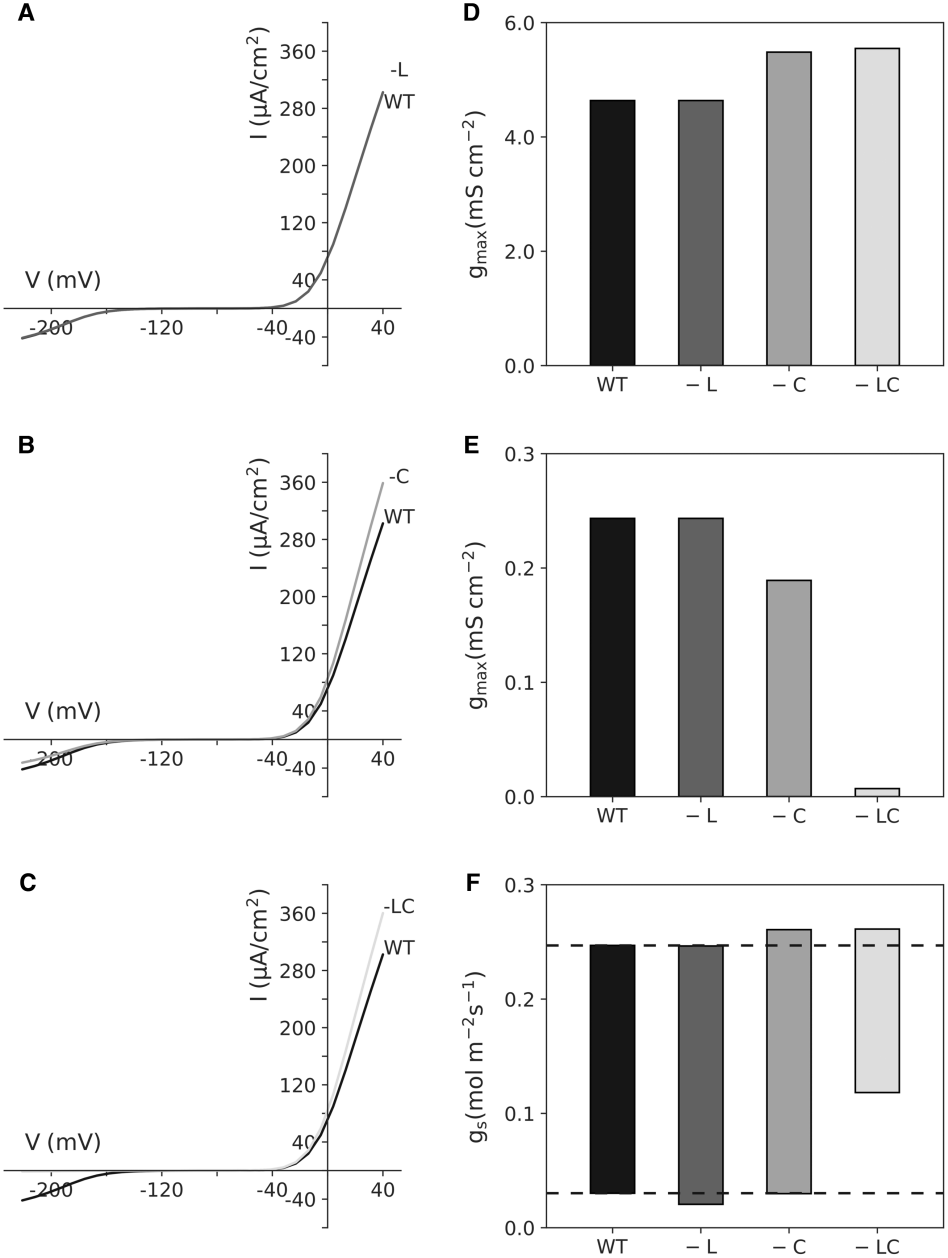
Deconstruction of the *Arabidopsis ost2* mutant in constitutive activation of AHA1, the dominant H^+^-ATPase of the guard cell plasma membrane. The OnGuard2 *Arabidopsis* model was used, as described by Wang et al. [8], without alteration (WT) and after eliminating the H^+^-ATPase dependencies on light (-L), on [Ca^2+^]_i_ (-C), and on both (-LC). (**A**–**C**) shows the steady-state current–voltage curves for the two K^+^ channel currents sampled at midday. The inward-rectifying K^+^ channel current is evident at voltages negative of −120 mV while the outward-rectifying current is visible at voltages positive of −60 mV. The WT curve (black lines) is reproduced in each frame for comparison with the *ost2* component deconstruction (grey lines). (**D**) Maximum conductance (*g*_max_) for the outward-rectifying K^+^ channel in (**A**–**C**) for each of the four-parameter combinations as determined by fitting to a Boltzmann function (see [72]). (**E**) Maximum conductance (*g*_max_) for the inward-rectifying K^+^ channel in (**A**–**C**) for each of the four-parameter combinations as determined by fitting to a Boltzmann function (see [72]). (**F**) Range of stomatal conductance (*g*_s_) calculated from OnGuard2 as described by Wang et al. [8] for each of the four-parameter combinations in (**A**–**C**). Dashed lines are included for reference to the WT model.

Consider the impact of mutating the plasma membrane H^+^-ATPase to render this pump constitutively active. Past studies have shown that two distinct mutations, *ost2-1* and *ost2-2*, result in constitutive activity of the AHA1 H^+^-ATPase, both largely insensitive to light and Ca^2+^ [70,71]. Since these parameters are easily accessed independently within the OnGuard platform, we encourage readers to trial the modelling platform, starting with the wild-type model provided and then manipulating the light- and Ca^2+^-dependencies for the plasma membrane H^+^-ATPase. We summarise in brief here the outcomes of manipulating each independently and together within OnGuard2. The model outputs (Figure 1) show that rendering the H^+^-ATPase active in both the light and dark has no substantive effect on either of the two dominant K^+^ channels (Figure 1A,D,E) or on stomatal conductance (Figure 1F). Eliminating the Ca^2+^ sensitivity of the pump in OnGuard2 promotes the outward-rectifying K^+^ channel and suppresses the inward-rectifying K^+^ channel (Figure 1B,D,E) but, similarly, has only a marginal effect on stomatal opening (Figure 1F). However, eliminating the parameter sensitivities to both light and Ca^2+^ strongly suppresses the inward-rectifying K^+^ channel and also has a substantial effect in elevating stomatal conductance in the dark (Figure 1F). These outputs highlight many separate behaviours of the guard cells and the associated stomatal conductance that appears counterintuitive. Among these, we may ask: (1) Why should the stomata of with guard cells incorporating the light-insensitive H^+^-ATPase open and close as in the wild-type model? (2) Why should the two Ca^2+^-insensitive formulations for the H^+^-ATPase affect the K^+^ channels? (3) What synergy between the two parameters, namely the insensitivities to light and Ca^2+^, gives rise to the enhanced stomatal conductance in the dark, despite the strong suppression of the inward-rectifying K^+^ channel current?

To the first of these questions, it is important to keep in mind that light activates other energy-dependent pumps, including the vacuolar VH^+^-ATPase and VH^+^-PPase [8,33,34]. Solute flux energised by the tonoplast pumps is equally important to generating the content for stomatal movements. Thus, as long as tonoplast transport remains under light-mediated control, it can be expected that stomatal conductance will follow a diurnal cycle similar to the wild-type, even if the plasma membrane H^+^-ATPase is light-insensitive. Indeed, reviewing the H^+^, K^+^, Cl^−^, and Mal fluxes across the tonoplast in these circumstances will show these are largely unaffected. Notably, at the end of the daylight period OnGuard2 yields substantial efflux of the osmotic solutes which, as these pass into the cytosol, lead to complementary fluxes across the plasma membrane. In short, the modelling shows that the trans effects of the substrate at the cytosolic face of the plasma membrane dominates to drive osmotic solute loss.

With regard to the second question, rendering the H^+^-ATPase insensitive to [Ca^2+^]_i_ leads to plasma membrane hyperpolarisation, as pump activity is no longer subject to this feedback control. Modelling shows that the enhanced H^+^-ATPase activity elevates pH_i_ and, by hyperpolarising the membrane, it also promotes Ca^2+^ influx through the inward-rectifying Ca^2+^ channels to elevate [Ca^2+^]_i_. The changes in [Ca^2+^]_i_ and pH_i_, in turn, suppress the inward-rectifying K^+^ channels while the elevated pH_i_ enhances the outward-rectifying K^+^ channels. These predictions are borne out experimentally and demonstrate an unexpected set of connections between K^+^ channel and H^+^-ATPase activity [8].

Finally, eliminating the sensitivities to both light and Ca^2+^ will be seen to enhance stomatal conductance in the dark through the combined impacts of the substantial elevations in nighttime pH_i_ and [Ca^2+^]_i_. Again, the changes in pH_i_ and [Ca^2+^]_i_ are a direct consequence of full H^+^-ATPase activity and its insensitivity to Ca^2+^, which together promote cytosolic alkalinisation unchecked by the concurrent Ca^2+^ influx through the inward-rectifying Ca^2+^ channels and [Ca^2+^]_i_ elevation, in this case also in the dark. Both the pH_i_ and [Ca^2+^]_i_ signals also suppress the inward-rectifying K^+^ channels and the alkaline pH_i_ enhances the outward-rectifying K^+^ channels [32]. These characteristics are borne out in experiments with the *ost2* mutants [7,8].

## Conclusion and outlook

Quantitative computational modelling is a gold standard for physiological analysis. These tools enable researchers to explore emergent properties associated with membrane transport and cellular signal processing. For stomata, the OnGuard platform encapsulates the mechanics of stomatal behaviour across scales from the molecular events of channel gating through to whole-plant transpiration. From an ecophysiological perspective, it bridges scales in a way that demonstrates the real potential for ‘reverse-engineering’ of efficiencies in whole-plant water use and carbon assimilation. The mechanics of membrane transport and metabolism in guard cells are marked by an extraordinary wealth of knowledge and quantitative kinetic detail at the cellular level that underpin their successful modelling. This knowledge can now be expanded to the challenges of foliar transpiration and carbon capture that previously were described empirically through quasi-linear relations with atmospheric humidity, CO_2_, and light, but without connection to guard cell mechanics. We expect the outcomes of modelling strategies, such as those we have outlined with OnGuard2, will prove central to guiding research and its application in the future.

## Perspectives

- The transport of osmotically active solutes across the membranes of stomatal guard cells drives stomatal movements, thereby regulating gas exchange for photosynthesis while protecting the leaf from excessive water loss via transpiration. The complexity of this transport makes mathematical modelling an essential tool, especially for understanding transport and its interactions that regulate gas exchange.
- Modelling strategies have commonly focused either on the molecular mechanics of transport and the associated metabolic processes or on the macroscopic properties of stomatal gas exchange in the leaf. Very few modelling efforts have sought to bridge these scales to interlock the molecular events in the guard cell to whole-plant gas exchange in the field, and only one platform incorporates the wealth of molecular kinetic detail needed to accurately describe guard cell membrane transport with true predictive power.
- Looking ahead toward future efforts to engineer crops for enhanced efficiencies in photosynthesis and water use, there is a clear need to bridge the molecular-macroscopic scales within a single, computational framework that is capable of spanning metabolic events including that of carbon assimilation.

## Abbreviations

RH: relative humidity
ROS: reactive oxygen species
